# A clinical practice guideline for the management of the foot and ankle in rheumatoid arthritis

**DOI:** 10.1007/s00296-024-05633-1

**Published:** 2024-06-08

**Authors:** Laura Ramos-Petersen, Andres Reinoso-Cobo, Ana-Belen Ortega-Avila, Jonatan Garcia-Campos, Jose-Antonio Bernal, Raquel Cantero-Tellez, Jose-Maria Martin-Martin, Rafael Caliz-Caliz, Sergio Tejero, Laura Cano-Garcia, Gabriel Gijon-Nogueron

**Affiliations:** 1 Department of Nursing and Podiatry, Faculty of Health Sciences, University of Malaga, Arquitecto Francisco Peñalosa 3, Ampliación de Campus de Teatinos, Malaga, 29071 Spain; 2 IBIMA, Malaga, Spain; 3 Department of Behavioral and Health Sciences, Miguel Hernández University, Alicante, Spain; 4 Institute for Health and Biomedical Research (ISABIAL), Alicante, Spain; 5 Department of Rheumatology, Hospital Marina Baixa, Villajoyosa(Alicante), Spain; 6 Department of Physiotherapy, Faculty of Health Sciences, University of Malaga, Arquitecto Francisco Peñalosa 3, Ampliación de Campus de Teatinos, Malaga, 29071 Spain; 7 Hospital Universitario Nuestra Señora de la Candelaria de Tenerife, Santa Cruz de Tenerife, Spain; 8 Rheumatology Department. Granada, Virgen de las Nieves Hospital, Granada, Spain; 9 Orthopaedic Department of University Hospital Virgen del Rocío. Head of Foot Ankle Unit, Department of Surgery, University of Sevilla, Seville, Spain; 10 Regional University Hospital of Malaga, Málaga, Spain

**Keywords:** Foot, Ankle, Arthritis, Rheumatoid, Care management, Patient

## Abstract

Rheumatoid arthritis causes progressive joint destruction in the long term, causing a deterioration of the foot and ankle. A clinical practice guideline has been created with the main objective of providing recommendations in the field of podiatry for the conservative management of rheumatoid arthritis. Thus, healthcare professionals involved in foot care of adults with rheumatoid arthritis will be able to follow practical recommendations. A clinical practice guideline was created including a group of experts (podiatrists, rheumatologists, nurses, an orthopaedic surgeon, a physiotherapist, an occupational therapist and patient with rheumatoid arthritis). Methodological experts using GRADE were tasked with systematically reviewing the available scientific evidence and developing the information which serves as a basis for the expert group to make recommendations. Key findings include the efficacy of chiropody in alleviating hyperkeratotic lesions and improving short-term pain and functionality. Notably, custom and standardized foot orthoses demonstrated significant benefits in reducing foot pain, enhancing physical function, and improving life quality. Therapeutic footwear was identified as crucial for pain reduction and mobility improvement, emphasizing the necessity for custom-made options tailored to individual patient needs. Surgical interventions were recommended for cases which were non-responsive to conservative treatments, aimed at preserving foot functionality and reducing pain. Moreover, self-care strategies and education were underscored as essential components for promoting patient independence and health maintenance. A series of recommendations have been created which will help professionals and patients to manage podiatric pathologies derived from rheumatoid arthritis.

**Supplementary Information:**

The online version contains supplementary material available at 10.1007/s00296-024-05633-1.

## Introduction

Foot involvement is very significant in rheumatoid arthritis (RA). Approximately 20% of patients report foot symptoms when their disease is diagnosed. As the disease evolves, this percentage increases to 90% [1–3]. In the initial stages, foot symptoms may go unnoticed in clinical assessments, and it is important to use physical assessment and radiological methods in the foot region to detect them [1, 4, 5].

The foot suffers joint destruction, increased ligament laxity and muscle-tendon dysfunction, modifying its biomechanics [1, 5, 6]. Consequently, RA negatively affects the quality of life, pain, function and stability of patients with RA, increasing their disability and making it difficult to carry out the usual tasks that require standing, carrying weight or walking long distances [3, 5, 7, 8].

To date, there are no Clinical Practice Guidelines (CPGs) that exclusively describe how pathologies affecting the foot and ankle should be addressed [9, 10]. The variability of clinical practice could lead to incorrect implementation of standardized protocols and misinterpretation of recommendations. This detrimentally affects patients through clinical errors, inadequate follow-up procedures, and treatment inaccuracies [11].

A CPG is a set of recommendations based on a systematic review of the evidence and the assessment of the risks and benefits of different alternatives, aiming to optimise the healthcare for patients. GPCs correspond to the first level of scientific evidence, with the function of helping professionals to make decisions, summarizing the evidence and transmitting confidence by being powerful instruments to reduce clinical variability [12].

Conservative intervention in the rheumatic foot should be part of the comprehensive evaluation of these patients, as well as to consult all the steps of the process in a CPG. The objective of this paper is to evaluate the efficacy of various interventions for managing foot and ankle issues in patients with RA, and to develop evidence-based recommendations that enhance patient care. Through a systematic literature review and Grading of Recommendations Assessment, Development and Evaluation (GRADE) analysis, this study aims to identify the most effective treatments for alleviating pain, improving functionality, and enhancing the quality of life for these patients, while also emphasizing the importance of tailored, patient-specific approaches in clinical practice.

## Methods

### Creation of the guide development group (GDG)

A multidisciplinary team was selected with the intent to get all relevant groups experienced with RA. The group was made up of experts in GPC methodology, health professionals and patients from different geographical areas (Malaga, Alicante, Tenerife, Granada and Sevilla, increasing the CPG value, dissemination and implementation. This is reflected by: five podiatrists, two rheumatologists, three nurses, one orthopaedic surgeon, one physiotherapist, one occupational therapist and four patients with RA. The multidisciplinary team was selected for its extensive experience in the management of RA and for its research career focused on RA.

The composition of the GDG is described below:

- *Coordination*: a specialist in foot rheumatology, as principal investigator (PI) and a specialist in CPG methodology coordinated the clinical and methodological aspects of the CPG.
- *Group of experts*: chosen for their qualities, experience and knowledge of RA. They were responsible for the development of the CPG recommendations.
- *Peer reviewers*: Methodological experts were tasked with systematically reviewing the available scientific evidence and developing the information that serves as a basis for the expert group to make recommendations.
- *Patients*: Two patients participated in the processing group and two patients participated as external reviews. All of them were patients from the Regional University Hospital of Malaga, Spain.

To ensure the optimal progress of the project, a detailed schedule was devised. None of the members of the group had conflicts of interest.

### Formulation of clinical questions

Initially, the scope and objectives of the guideline were collaboratively defined and unanimously agreed upon by all members of the GDC, including experts, patients, and through reference to scientific literature. The formulation of clinical questions followed a consensual approach, ensuring a structured format aligned with the Patient, Intervention, Comparison, and Outcome (PICO) framework [13]. This not only enhances the scientific rigor but also facilitates the formulation of recommendations. Patient involvement remained integral throughout the entirety of the process. Subsequently, upon reaching consensus on the proposed objectives, clinical questions were established to address these objectives, as outlined in Table 1. Preferably, we sought to answer these questions through systematic reviews and meta-analyses.

Finally, the PICO questions that were answered were:

- What is the role of the implementation of foot care in improving health, the ability to move autonomously, independence and functionality, and improving quality of life in patients with RA compared to not implementing foot care?
- What is the most commonly used foot care in RA patients with foot and ankle involvement?
- What indicators suggest progression of foot and ankle involvement in RA disease? How to improve foot and ankle symptoms in RA?
- How to implement foot care in patients with RA?
- How to reduce variability in foot care in patients with RA?

**Table 1:** GEG research questions for systematic reviews

Question	Patient diagnosed with RA	Intervention	Comparison	Outcome
Role of foot care implementation?		• Foot care	• Non-implementation of care	• Improved health
				• Improved autonomous travel capability
				• Improved independence
				• Improved functionality
				• Improved quality of life
What is the most frequent and common foot care?		• Footwear recommendations		
		• Debridement of hyperkeratosis and enucleation of helomas (chiropody)		
		• Musculoskeletal problems		
		• Deformities of the foot		
		• Ulcers		
What are the indicators that foot and ankle damage is advancing?				Indicators of progression
How to improve foot and ankle symptoms?		• Interventions	• Pharmacological alternatives	• Improvement of symptoms in the foot and ankle
How to effectively implement foot care?		• Chiropody • Footwear • Foot orthoses • Surgery • Self-care • Ulcer Management • Physical therapy • Injections	• Pharmacological alternatives	• Adherence to treatments, including strategies, protocols, referral circuits
How to reduce variability in foot care?				• Reduce variability

### Literature search, evaluation and synthesis of evidence

A systematic review of the scientific literature was carried out and in cases where there was no scientific literature, a Delphi survey was carried out [12].

The search was carried out in the databases of Medline (through Pubmed), SCOPUS, CINHAL, PEDro using the following terms: rheumatoid arthritis, callus, corn, hyperqueratosis, nails, foot, forefoot, feet, ankle, ankle joint, joint, footwear, shoe*, boot*, deck, trainer*, sneaker, orthoses, orthosis, insole, plantar, bones of lower extremity, Hallux, first metatarsophalang*, surgic*, non-conservative treatment, pain, disab*, funct*, foot lesions, ulcer, skin lesions, glucocorticoids, triamcinolone, hyaluronic acid, viscosupplements, platelet-rich plasma, injections, intra-articular. Free language terms and descriptors were combined to balance the sensitivity and specificity of the searches (Annex 8). No time restrictions were set. The search ended in June 2022, and was limited to human studies, written in either Spanish or English.

### Inclusion and exclusion criteria

Studies were included if they presented the following characteristics.

- *Population*: Adult patients (over 18) diagnosed with RA and/or diagnosed according to the 2010 RA criteria approved by the American College of Rheumatology and European League Against Rheumatism [14].
- *Intervention*: conservative and non-conservative foot care.
- *Outcome variables*:
- **Primary Outcome - Improvement in Foot Health**: Reduction in foot pain as measured by the Foot Function Index (FFI) or Visual Analog Scale (VAS) scores.
- **Secondary Outcomes: Improved Health**: Lower overall RA disease activity scores, such as the Disease Activity Score 28 (DAS28), indicating better systemic health. **Autonomous Movement**: Increased ability to perform daily activities without assistance, measured by the Health Assessment Questionnaire Disability Index (HAQ-DI). **Independence**: Enhanced self-sufficiency in personal care and mobility tasks, potentially assessed through patient-reported outcome measures. **Functionality**: Improved physical function, specifically in the lower extremities, measured by the Lower Extremity Functional Scale (LEFS). **Quality of Life**: Higher scores on the Rheumatoid Arthritis Quality of Life (RAQoL) questionnaire or the Short Form Health Survey (SF-36). **Symptoms**: Reduction in specific RA symptoms such as joint swelling, stiffness duration in the morning, and the number of tender/swollen joints. **Adherence to Treatments**: Higher rates of compliance with prescribed medication and non-pharmacological interventions, monitored through patient self-reports or pharmacy refill rates. **Reduced Variability**: Decreased diversity in treatment approaches among healthcare providers, indicating standardization of care practices, assessable through review of medical records or surveys.
- *Study design*: systematic reviews or meta-analyses and randomized clinical trials, observational studies and case-control studies.

Studies which included people under 18 years of age or pregnant women, conference abstracts, posters, narrative reviews, letters, and any type of unpublished study were excluded.

### Assessment of the quality of the studies

Two members of the GDG assessed the risk of bias of the individual studies. The Cochrane Handbook for Systematic Reviews of Interventions was used [15] to evaluate randomised controlled trials (RCTs) and the Newcastle-Ottawa scale (NOS) [16] for observational studies. NOS is a valid tool for assessing the quality of any observational design that has an adapted version [17, 18], which assesses selection bias, performance bias, detection bias, and reporting bias.

Each RCT was assessed to consider if there was any bias related to the following domains: the randomisation process; deviations from interventions; lack of outcome data; outcome measurement and selection of reported outcome. In addition, the Preferred Reported Items of Systematic Reviews and Meta-analysis (PRISMA) was used to evaluate the systematic reviews and meta-analyses [19].

### Formulation of recommendations

In formulating recommendations, careful consideration was given to the quality, quantity, and consistency of scientific evidence, as well as their applicability and clinical implications. Any contentious or unsupported recommendations were addressed through consensus during a dedicated meeting of the GDG.

In order to empower patients and their families in making informed decisions regarding their healthcare, specific educational materials have been incorporated. These materials, available in Annex 9, are designed to provide comprehensive information tailored to the needs and understanding of patients and their families. By offering accessible resources, the aim is to enhance communication between healthcare providers and patients, fostering a collaborative approach to treatment and care.

### GPC external review

An external review of the guideline was carried out by professionals selected for their knowledge of RA and methodology used to create guidelines. The ultimate goal was to increase the external validity of the document and ensure the accuracy of its recommendations.

In addition, OpenReuma contributed to the process of public exposure and dissemination. The present guide was available on the OpenReuma website along with a form for collecting statements. OpenReuma is a non-profit scientific association that brings together healthcare professionals with an interest in rheumatology.

### Evaluation and synthesis of the evidence

The quality of evidence was evaluated utilizing the rigorous methodology established by the GRADE group. Recommendations underwent a voting process, scored on a scale of 0 to 10, with those averaging ≥ 6 among the GDG advancing to the next stage of the CPG. Recommendations scoring below 6 points underwent thorough deliberation to determine their inclusion or exclusion from further consideration [20].

Through the Guidelines Development Tool (http://gdt.guidelinedevelopment.org/*)*, confidence was assessed to support the recommendations developed. As a result, each recommendation received a rating: high, moderate, low, and very low [21, 22].

## Results

For a better understanding and dissemination of the recommendations, the answers have been divided into the different treatments: chiropody, footwear, foot orthoses, surgery, self-care, ulcer management, physical therapy, and injections.

All recommendations, except physical therapy, were developed following the results from a systematic literature review and a subsequent analysis using the GRADE system for drafting the recommendations. In instances where scientific literature was unavailable, a Delphi survey was conducted among the members of the GDC to gather expert consensus.

Our findings reveal varied levels of evidence across different treatment modalities for foot and ankle management in patients with RA. This section delineates the quality of evidence and the strength of recommendations derived from the analysis.

1. **Chiropody**: The GRADE analysis indicates a very low level of evidence regarding the efficacy and outcomes of chiropody interventions (Annex 1).
2. **Footwear**: The assessment of therapeutic footwear, including both custom and standard options, yielded a moderate to very low grade of evidence (Annex 2).
3. **Foot Orthoses**: The evidence supporting the use of foot orthoses was rated as moderate (Annex 3).
4. **Surgery**: Surgical interventions for foot and ankle issues in rheumatoid arthritis patients were found to have a very low grade of evidence (Annex 4).
5. **Self-Care**: The effectiveness of self-care strategies, including education on foot health and routine care practices, was assessed to have a very low level of evidence (Annex 5).
6. **Ulcer Management**: Similarly, the GRADE assessment for ulcer management strategies yielded a very low level of evidence (Annex 6).
7. **Injections for Joint and Tendon**: The evaluation of the efficacy of injections, specifically in joints and tendons in the foot and ankle, also received a very low evidence grade (Annex 7).

The recommendations are as follows:

### Chiropody recommendations

Are chiropodies recommended for patients with RA? Do chiropodies need to be complemented with other treatments?

Hyperkeratotic lesions on the feet cause pain and disability to patients despite being considered a minor problem [23]. 65% of patients with connective tissue diseases present plantar callosities, and this percentage increases in patients with RA due to the alterations produced by the disease itself [24, 25]. When these hyperkeratoses are left untreated, they may cause some deeper damage to the tissues, which could lead to tissue ulceration [24].

Toe deformities, presence of hallux valgus and flat feet are risk factors associated with the appearance of hyperkeratosis, which can cause pain [24–26]. These risks factors are frequently present in patients with RA. These patients have pressure peaks which are higher than normal values, with an atypical distribution of pressures and forces acting on the foot [27].

Chiropody is known as the mechanical debridement of thickened skin with a scalpel blade, being the most common treatment for painful hyperkeratotic lesions [28]. Debridement decreases focal pressures, reducing the appearance of ulcers and significantly facilitating their healing [29]. In addition, in the short term, there is a pain decrease and an improvement in functionality after treatment, which is related to an increase in gait speed, cadence, stride length and bipodal support time in patients with RA [29].

However, it should be noted that isolated debridement of painful hyperkeratoses in the forefoot should not be used, even if it reduces pain in the short term [29]. This technique should be combined with the use of appropriate footwear and foot orthoses in patients with RA [28, 29].

Therefore, it should be concluded that chiropodies are recommended for the removal of hyperkeratotics, thus achieving a reduction in pain in the short term and an improvement in functionality. In addition, they should be complemented by orthopedic treatments, including appropriate footwear and foot orthoses (Table 2).

**Table 2:** Recommendations

Chiropody recommendations and GRADE (Annex 1)	
Chiropodies are recommended for the removal of hyperkeratotic (helomas and thylomas) and nail lesions	Very low
Chiropodies should be completed with orthopaedic treatments or orthopaedic footwear	Very low
Footwear recommendations and GRADE (Annex 2)	
Standardized therapeutic footwear benefits patients with RA by reducing pain and improving physical functionality, compared to store-bought footwear	High
Standardized therapeutic footwear provides benefits in foot functionality (reducing plantar pressure), foot pain, physical functionality, and quality of life	High
Foot orthoses recommendations and GRADE (Annex 3)	
Foot orthoses optimize the biomechanics and function of the foot, providing cushioning and unloading the structures of the foot	High
Foot orthoses reduce foot pain, improve physical function and quality of life	High
Custom foot orthoses reduce foot pain and functionality, balance, and quality of life	High
Surgical recommendations and GRADE (Annex 4)	
Total ankle arthroplasty is recommended for patients with RA	Very low
Arthrodesis of the 1^st^ metatarsophalangeal joint and arthroplasty of the 2nd to 5th metatarsal heads are recommended	Very low
Scarf’s technique is positive in short-term correction of hallux abductus valgus	Very low
Self-care recommendations and GRADE (Annex 5)	
It is recommended to work with patients on the limiting factors for self-care with all the factors involved in the care of patients with RA	Very low
Ulcer management recommendations and GRADE (Annex 6)	
Care of skin ulcers, such as vascular ulcers with a high risk of infection, is recommended	Very low
Physical therapy recommendations and GRADE	
Moderate physical exercise with limited supervision is advisable as long as intensity, frequency and appropriate duration is respected	Expert opinion
Supervised in-office physical exercise has positive effects on quality of life, physical functioning, and pain	Expert opinion
Injections recommendations and GRADE (Annex 7)	
Corticosteroid injection with previous ultrasound information improves stiffness and physical function results compared to infiltration with clinical and radiographic data alone	Moderate evidence
Corticosteroid injection with clinical and radiographic data alone is capable of improving pain and, to a lesser extent, stiffness and physical function	Moderate evidence
In patients with ankle arthritis, an injection of triamcinolone hexacetonide corticosteroid is effective in reducing pain and inflammation	Low evidence
In patients with RA and tendinitis of the foot, an injection of corticosteroids together with a podiatric-orthotic program is effective in terms of pain, function and ultrasound (Doppler)	Moderate evidence

### Footwear recommendations

What are the benefits of therapeutic footwear for patients with RA?

The choice of footwear might be an issue for RA patients. Therapeutic footwear includes custom-made and off-the-shelf footwear. Custom-made therapeutic footwear is made for an individual patient based on individual measurements and specifications, so a variety of technical adaptations can be incorporated; while ready-made therapeutic footwear includes mass-produced shoes with greater depth, support, or technical adaptations [30]. When patients with RA wear therapeutic footwear, an improvement in pain and mobility has been demonstrated as these patients present complex needs due to their pain and structural changes to their feet. Due to the deformity of the patients, standard footwear can cause pressure areas due to poor fit, whereas orthopedic footwear is designed to allow space for these deformities, reducing pressure, which can lead to pain, skin lesions, and ulcers. However, patients with RA, particularly women, often find therapeutic footwear unacceptable due to aesthetics, price, or limited availability. This situation often leads to dissatisfaction when choosing and wearing footwear. Their social behaviour might be altered, causing a negative impact on body image and emotions due to their footwear [31, 32].

Patients who decide to wear standardized footwear can do so by looking for a more aesthetic alternative to therapeutic footwear. However, standardized or ready-made footwear may not be suitable and can exacerbate their foot problems, creating an issue when wearing foot orthoses [32, 33].

Randomized clinical trials and meta-analyses indicate that therapeutic footwear benefits RA patients in terms of pain reduction and improvement in physical function, compared standard footwear. In addition, standardized therapeutic footwear provides benefits in foot functionality, foot pain, physical functionality, walking speed, stride length, and quality of life [34, 35] (Table 2).

### Foot orthoses recommendations

What are the effects of foot orthoses for patients with RA?

Foot orthoses are an important conservative treatment option for RA-related foot problems and are frequently prescribed in clinical practice [36]. They are placed inside the shoe to control the movement of the foot when walking to limit pain and deformity of the foot, modifying the neuromuscular and skeletal system [36, 37]. Its main objectives for patients with RA is to reduce pain and disability, improving the patient’s quality of life [38].

The efficacy of foot orthoses in patients with RA has been confirmed, including both personalized orthoses, which are specifically tailored to patients, and standardized orthoses [39, 40]. Its efficacy may be influenced by shorter duration of illness, younger age, and pain and disability values [41].

Early and ongoing interventions with foot orthoses provide a significant reduction in foot pain in the short term, with a reduction in disability and improved long-term foot health outcomes. Early intervention has demonstrated a pain reduction within the first 3 months of use and with a small additional symptomatic improvement up to 6 months [42]. It has been suggested that this early management helps avoid or delay late-stage orthopaedic surgery [43]. With these interventions, a window of opportunity is generated in early stage RA with the aim of minimizing foot deformation before irreversible joint damage occurs [41, 43–45].

Foot orthoses vary widely in their material, design, and manufacturing method. This variation is further increased by additional elements such as posts, wedges, and cushioning [46]. It has been concluded that foot orthoses made from soft materials can reduce plantar pressure of the forefoot compared to semi-rigid materials [47], while other studies concluded that rigid and semi-rigid materials in custom foot orthoses reduce rearfoot pain among patients with RA [48]. Therefore, more high-quality, better-designed studies with more specific parameters are needed (Table 2).

### Surgical recommendations

Which foot surgeries are recommended for patients with RA?

Osteoarticular surgical treatment of the foot should allow the reduction of deformity, pain and preservation of functionality in patients with RA. The indication for surgery is mainly due to pain, which has not improved with conservative treatment and reduces the patient’s quality of life. The intention of surgery is not curative, since the degenerative evolution of the disease will continue to progressively deteriorate the rest of the joints.

The surgical technique will depend on the deformity, age and bone quality, as well as the degree of joint destruction [49]. The surgical techniques most commonly described in the literature are arthroplasty, arthrodesis and osteotomy of the metatarsophalangeal joints (MTP) and total ankle arthroplasty [50].

Short-term studies (6–12 months) report an improvement of foot pain [51], but long-term studies report increased pain and recurrence of deformities [52]. In most studies, the follow-up period is insufficient despite satisfactory results.

Surgeries of the MTP joints and toes have been reported most frequently [52, 53], coinciding with the prevalence of foot deformities in RA, having a high frequency of hallux valgus, hallux rigidus, and claw toes [54, 55].

First MTP joint arthrodesis [51, 56, 57] and second to fifth MTP joint arthroplasty [58], are the most documented surgeries [26].

Post-surgical iatrogenic effects have also been described, such as: ankylosis in the midfoot [59], increase in rearfoot varus, and increased forefoot stiffness, failed total ankle arthroplasty leading to arthrodesis of the ankle [60]. However, in a recent meta-analysis, it has been concluded that total ankle replacement is a safe procedure for RA patients with difficulties close to other reasons for ankle replacement [61] (Table 2).

### Self-care recommendations

Is self-care recommended for patients with RA?

Self-care is the strategy that should be carried out to cope with life events and stressors that can have a negative impact on health, with the aim of alleviating the symptoms of the disease, promoting good health, achieving the independence of the patient [62].

Self-care, therefore, is a regulatory function that patients acquire through health education carried out by health professionals which may be influenced by different factors: social support, demographic characteristics, knowledge of the disease, and physical function [63].

It should be taken into consideration that the patient’s ability to carry out self-care can vary dependant on: age, illness, health education, health status, physical condition, and perceived pain [64].

Patients with RA may have difficulties in carrying out their self-care due to the disability caused the evolution of the disease, therefore, self-care of the feet must be encouraged from the beginning of the disease, being incorporated into the patient’s usual tasks. Foot care is important to prevent and maintain their health, promoting independence, mobility and personal and social activity. It is essential that the patient is able to identify problems in their feet, as well as having the knowledge and skills to treat them [65].

Therefore, it has been concluded that daily hygiene, skin and nail care, the use of appropriate footwear and socks, the use of toe spacers if necessary, the use of foot orthoses and specific exercises for the lower limb are included as self-care of the feet [66] (Table 2).

### Ulcer management recommendations

Is it recommended the care of skin ulcers in patients with RA?

The management of foot problems in patients with RA may involve a variety of interventions, such as treatment of skin lesions resulting from ulcers [67]. Previous studies established that ulceration has been more commonly observed in female RA patients with prolonged disease, with many patients presenting multiple episodes of ulceration and not always in the same site [68].

As time passes and the disease progresses, foot deformity and trauma caused by footwear can increase the risks of damage to the surrounding skin, resulting in loss of skin integrity and can lead to foot ulcers [69]. The overall prevalence of foot ulceration in patients with RA is between 10 and 13% [69, 70], with an added impact on their quality of life [70, 71].

Regarding the location of ulcers, more than 50% are located at the toes, and 15% at the rearfoot, the most common place being on the dorsal aspect of hammertoes [68], followed by the plantar side of the metatarsal heads [70, 71]. Increased age and duration of the disease increases the risk of ulcers [69]. In addition, patients who have undergone treatment with targeted therapies are at increased risk of infection, causing skin fragility and hindering tissue repair [69, 70] (Table 2).

### Physical therapy recommendations

Which type of physical exercise is recommended for patients with RA?

Non-pharmacological treatment modalities are often used together with pharmacological treatment in patients with RA [72]. There are different modalities of physical therapy, including physical exercise, which are used in RA to generate therapeutic physiological effects with the aim of reducing pain or restoring function [73].

Physical exercise can be considered as part of physical therapy. It is necessary to practice exercise supervised by a professional if the patient presents with any limitations in activities of daily living or if the patient is unable to achieve an adequate level of physical functioning independently. Therefore, it is recommended to perform moderate physical exercise with limited supervision as long as the intensity is respected and frequency and duration is adequate for each patient [74–76].

Although there are currently no specific recommendations for foot physical exercises for individuals with foot and ankle osteoarthritis (OA) [77], exercise remains a promising intervention for improving outcomes related to RA and alleviating negative emotional states [78].

Effective exercise interventions induce physiological responses such as increased flexibility, muscular strength, and cardiovascular fitness. This efficacy is contingent on the appropriate intervention, correct dosing, and patient adherence [79]. Cardiovascular training for individuals with RA should involve no-impact sports such as Nordic walking, dancing, cycling, or water-based exercises [78]. The recommended intensity ranges from moderate to vigorous, with high-intensity training—up to 90% of the predicted maximal heart rate or 80% of one-repetition maximum—proving effective and feasible for people with RA [80].

In studies on foot and ankle OA, muscle strength, kinetics, and kinematics are objective measures of function often specified as secondary outcomes [81–84]. Therefore, exercises focusing on foot stretching, strengthening, proprioception, and flexibility may be beneficial for patients with foot and ankle OA. However, there is limited evidence on the effects of flexibility exercises, and almost no literature evaluates neuromotor exercises [78].

According to Shamus J et al. [81], incorporating sesamoid mobilization, flexor hallucis longus strengthening and gait training to a physical therapy program for first MTP joint OA, can significantly reduce pain intensity. Additionally, foot and ankle exercises could be an effective treatment not only for improve local pain on foot and ankle, but also could be a strategy for improving pain and functional deficits in individuals with patellofemoral pain [85].

In a meta-analysis is has been confirmed that strength exercises reported a positive effect on pain in the short and medium term, but it did not increase the aerobic capacity of the patients. On the other hand, they concluded that aerobic exercise improved the capacity of the patient to practice physical activity, but it did not report a positive nor negative effect in pain. Therefore, the most important thing is to consider the evolution of the patient and their symptoms, creating personalized exercise program [86].

Electrotherapy is used to control pain and increase muscle strength and function. One form of electrotherapy that is often used is transcutaneous electrical nerve stimulation (TENS), which can help relieve pain [87].

Thermotherapy consists of the local application of cold or heat in isolation or through contrast baths with immersion in hot and cold water [88]. However, the effects of contrast baths may be more beneficial than applying cold during acute phases of pain [88]. Dry or water-jetted local heat can be used to provide short-term pain relief and to decrease joint stiffness, while paraffin wax baths provide longer-term results according to the British Society of Rheumatology (BSR) and the British Health Professionals in Rheumatology (BHPR) [72]. However, paraffin baths should be avoided in patients presenting with an active RA flare-up [73].

The warming effects of continuous ultrasound can also reduce muscle spasms and stimulate blood flow to help decrease inflammatory toxins [89].

Low-level laser therapy is another modality for relieving pain and improving function in patients with RA. The laser emits a single wavelength of pure light, which causes a photochemical reaction within the cells and can have an effect on flexibility and pain [90] (Table 2).

### Injection recommendations

Which are the effects of corticosteroid injection for patients with RA?

Intra-articular corticosteroid injections are frequently used in the treatment of RA throughout the lower limb, although with the greatest evidence of efficacy confined to the knee joint. Meta-analyses have shown a positive short-term therapeutic effect, which may be mistaken for a placebo effect. Corticosteroid injections are capable of improving pain, and to a lesser extent, stiffness and physical function. This has been concluded after the analysis of clinical and radiographic data [91]. In addition, these injections are effective in the treatment of tenosynovitis, especially if combined with foot orthoses, improving pain levels and functionality of the foot [92].

Corticosteroid injection, specifically triamcinolone hexacetonide, is effective at reducing pain and inflammation [93]. In pharmacokinetic studies, corticosteroids with more microcrystalline properties have a longer half-life at the joint level [94]. An improvement in pain at rest after ankle injections has been described in patients with RA, and the injection of corticosteroids significantly improves pain, oedema and morning stiffness [93, 95].

Although corticosteroid injection is a method that significantly alleviates local inflammation, its adverse effects, such as local deterioration in repeated injections, should also be highlighted. Another intra-articular therapy could be the injection of hyaluronic acid, which has shown improvement in short-term foot function and reduction of pain [96, 97].

The use of anatomical landmarks for needle placement in injections is not always reliable and unanimous for all patients. One study predicted that one-third of ankle joint injections result in extra-articular localization [98]. Therefore, the use of ultrasound to perform injections is an essential aspect for the effect to occur in the desired place. The use of this technique improves results in the short term, with even greater success in the long term, enough to justify the additional cost of using imaging [99]. Previous studies indicate that after the injection of corticosteroids in patients with RA and foot pain, with and without ultrasound, the information provided by ultrasound helps to obtain better results in relation to the physical function [100] (Table 2).

## Discussion

This study contributes to the field of rheumatology by presenting a comprehensive set of CPGs dedicated to the management of foot and ankle pathologies in RA patients. These guidelines fill a critical gap in the literature, offering detailed, evidence-based recommendations where previously there was a notable lack of specificity and depth, particularly in the context of non-pharmacological treatments. Consequently, recommendations that will help professionals and patients to manage podiatric pathologies derived from RA have been created.

The first joints affected by the disease are the small synovial joints, with the foot being one of the body regions with the highest incidence. Delayed management of the alterations causes a loss in foot functionality, which causes high levels of pain and increased disability, which ultimately translates into a decrease in the quality of life of the RA patient [1, 5, 6].

Currently, the pharmacological treatment of RA is chronic and modifiable, with drug doses being periodically adjusted according to the level of disease activity. Therefore, starting RA treatment as soon as possible is very important, ideally within the first 12 weeks from the onset of symptoms [101–103]. Likewise, it is vital to establish non-pharmacological treatment of the foot as soon as possible, but there are no evidence-based guidelines that health professionals can follow in this regard. This means that it is not possible to follow protocols that guide foot management in patients with RA. Some CPGs are available that offer recommendations to rheumatologists and other healthcare professionals involved in the care of RA patients in a holistic way, where some foot-related recommendations are available, but not focused on feet and lacking in depth [9, 10].

The importance of reducing the variability of clinical practice is mainly to avoid negative repercussions on the patient due to clinical misinterpretations, which may be related to inexperience, errors in the collection of data in medical records, and/or lack of resources and updating of knowledge of specialist professionals [11].

As strengthen, the creation of the current CPG presents a great advantage in the management of foot and ankle problems in patients with RA, being the first guide focused exclusively on the foot and ankle in RA. In addition, it should be noted that the perspective of patients with RA has been considered in the development of the CPG, both in the guideline group when developing the questions and in the review of the final manuscript. In this way, it is intended to improve the health, the ability to move autonomously, independence and therefore the functionality and quality of life of people affected by RA, with foot and ankle involvement, by establishing recommendations with high evidence. In addition, to reduce the variability in clinical practice among professionals in the diagnosis and treatment of foot and ankle involvement in patients with RA; to monitor the progress of foot and ankle involvement with the aim of carrying out preventive and early treatment and, finally, to improve the approach to foot and ankle problems in patients with RA, promoting rationality and efficiency in the choice of different treatments.

It is also necessary to point out the limitation that this guide presents in relation to the scarcity of articles of high evidence to answer some of the questions initially proposed. This was solved by recruiting a role of experts who contributed with their clinical and research experience.

In the process of developing this guide, some priority areas for future research have been identified:

- Effects of physical exercise on the feet of RA patients, including their level of pain, disability, and quality of life.
- Clinical trials in deficit areas such as foot surgery, patient self-care or chiropodies.
- To work to educate the health care professionals involved in the foot therapies of these patients.

With the recommendations presented in this paper, professionals will be better guided through the best management strategies for the foot and ankle pathologies of the RA patient. Furthermore, the information outlined in this guide can be used to generate information which can be passed onto RA patients.

## Conclusion

Our study culminates in evidence-based recommendations for foot and ankle management in rheumatoid arthritis patients, highlighting the utility of chiropody, foot orthoses, and therapeutic footwear in reducing pain and enhancing mobility. Custom solutions are particularly emphasized for their role in improving quality of life. Surgical options are advised for cases refractory to conservative measures, aiming to preserve functionality. The importance of self-care and education in promoting independence is also underscored. These guidelines serve as a foundation for clinicians, emphasizing the need for ongoing research to refine and update therapeutic strategies.

### Electronic supplementary material

Below is the link to the electronic supplementary material.

Supplementary Material 11

Supplementary Material 12

Supplementary Material 13

Supplementary Material 14

Supplementary Material 15

Supplementary Material 16

Supplementary Material 17

Supplementary Material 18

Supplementary Material 19
