# Supplementary material for: A clinical practice guideline for the management of the foot and ankle in rheumatoid arthritis
Source: Rheumatol Int. 2024 Jun 8;44(8):1381–93. doi: 10.1007/s00296-024-05633-1 (PMC11222212; doi:10.1007/s00296-024-05633-1)
Supplement: Supplementary file 1 — Supplementary Material 11 [file 296_2024_5633_MOESM11_ESM.docx]

## Annex 1. GRADE Chiropody Assessment

**Question:** Chiropody in Rheumatoid Arthritis Patients

| **Certainty assessment** | | | | | | | **No. of patients** | | **Effect** | | **Certainty** | **Importance** |
| --- | --- | --- | --- | --- | --- | --- | --- | --- | --- | --- | --- | --- |
| **No. of studies** | **Study Design** | **Risk of bias** | **Inconsistency** | **Indirect Evidence** | **Imprecision** | **Other Considerations** | **Chiropody in patients with RA2** |  | **Relative(95% CI)** | **Absoluto(95% CI)** |  |  |
| **Foot Pain Reduction with Chiropod Treatment** | | | | | | | | | | | | |
| 2 | Randomised trials | Serious | Serious | Serious | Serious | None | 52/52 (100.0%) |  | Not Estimable |  | ⨁◯◯◯Very low |  |

**Bibliography:**

1. Davys HJ, Turner DE, Helliwell PS, Conaghan PG, Emery P, Woodburn J. Debridement of plantar callosities in rheumatoid arthritis: A randomized controlled trial. Rheumatology. 2005; 44(2):207–10.

2. Siddle HJ, Redmond AC, Waxman R, Dagg AR, Alcacer-Pitarch B, Wilkins RA, et al. Debridement of painful forefoot plantar callosities in rheumatoid arthritis: The CARROT randomised controlled trial. Clin Rheumatol. 2013; 32(5):567–74.
