# Supplementary material for: A clinical practice guideline for the management of the foot and ankle in rheumatoid arthritis
Source: Rheumatol Int. 2024 Jun 8;44(8):1381–93. doi: 10.1007/s00296-024-05633-1 (PMC11222212; doi:10.1007/s00296-024-05633-1)
Supplement: Supplementary file 2 — Supplementary Material 12 [file 296_2024_5633_MOESM12_ESM.docx]

## Annex 2. GRADE Footwear Assessment

**Question: Therapeutic** footwear in RA patients

| **Certainty assessment** | | | | | | | **No. of patients** | | **Effect** | | **Certainty** | **Importance** |
| --- | --- | --- | --- | --- | --- | --- | --- | --- | --- | --- | --- | --- |
| **No. of studies** | **Study Design** | **Risk of bias** | **Inconsistency** | **Indirect Evidence** | **Imprecision** | **Other Considerations** | **Therapeutic footwear** | **Non-therapeutic footwear** | **Relative(95% CI)** | **Absoluto(95% CI)** |  |  |
| **Biomechanical compensation of the foot by means of orthopaedic footwear.** | | | | | | | | | | | | |
| 5 | Randomised trials | Serious | It's not serious | It's not serious | It's not serious | None | 125/248 (50.4%) | 123/248 (49.6%) | Not Estimable |  | ⨁⨁⨁◯Moderate |  |
|  | | | | | | | | | | | | |
| 6 | Observational studies | Very serious | It's not serious | It's not serious | It's not serious | None | 292/292 (100.0%) |  | Not Estimable |  | ⨁◯◯◯Very low |  |

**Bibliography:**

1) Dahmen R, Buijsmann S, Siemonsma PC, Boers M, Lankhorst GJ,Roorda LD) Use and effects of custom-made therapeutic footwear onlower-extremity-related pain and activity limitations in patients withrheumatoid arthritis: A prospective observational study of a cohort. JRehabil Med 46(6):561–567. 2014

2) Park C, Craxford AD Surgical footwear in rheumatoid arthritis—a patient acceptability study. Prosthet Orthot Int 5(1):33–36. 1981

3) Pullar T, Anderson M, Capell HA, Millar A Comfort shoes–a cheaper alternative to surgical shoes in rheumatoid arthritis. Health Bull41(5):258–262, 1983.

4) Chalmers AC, Busby C, Goyert J, Porter B, Schulzer M Metatarsalgia and rheumatoid arthritis—a randomized, single blind, sequential trial comparing 2 types of foot orthoses and supportive shoes. J Rheumatol 27(7):1643–1647, 2000.

5) Cho NS, Hwang JH, Chang HJ, Koh EM, Park HS Randomized controlled trial for clinical effects of varying types of insoles combined with specialized shoes in patients with rheumatoid arthritis of the foot. Clin Rehabilit 23(6):512–521, 2009.

6) Fransen M, Edmonds J Off-the-shelf orthopedic footwear for peoplewith rheumatoid arthritis. Arthritis Care Res 10(4):250–256, 1997.

7) Hennessy K, Burns J, Penkala S Reducing plantar pressure in rheumatoid arthritis: a comparison of running versus off-the-shelf orthopaedic footwear. Clin Biomech 22 (8):917–923, 2007.

8) Williams AE, Rome K, Nester CJ A clinical trial of specialist footwear for patients with rheumatoid arthritis. Rheumatology 46 (2):302–307. 2006

9) Barrett JP Jr Plantar pressure measurements. Rational shoe-wear in patients with rheumatoid arthritis. JAMA 235(11):1138–1139. 1976

10)Bagherzadeh Cham M, Ghasemi MS, Forogh B, Sanjari MA, Zabihi Yeganeh M, Eshraghi A Effect of rocker shoes on pain, disability and activity limitation in patients with rheumatoid arthritis. Prosthet Orthot Int 38(4):310–315. 2014

11) Moncur J, Ward JR (1990) Heat-Moldable Shoes for Management of Forefoot Problems in Rheumatoid. Arthritis Arthitis Care Res 3(4):222–226.
