# Supplementary material for: A clinical practice guideline for the management of the foot and ankle in rheumatoid arthritis
Source: Rheumatol Int. 2024 Jun 8;44(8):1381–93. doi: 10.1007/s00296-024-05633-1 (PMC11222212; doi:10.1007/s00296-024-05633-1)
Supplement: Supplementary file 3 — Supplementary Material 13 [file 296_2024_5633_MOESM13_ESM.docx]

## Annex 3. GRADE Foot Orthoses Assessment

**Question: Plantar** Orthoses in Rheumatoid Arthritis Patients

| **Certainty assessment** | | | | | | | **No. of patients** | | **Effect** | | **Certainty** | **Importance** |
| --- | --- | --- | --- | --- | --- | --- | --- | --- | --- | --- | --- | --- |
| **No. of studies** | **Study Design** | **Risk of bias** | **Inconsistency** | **Indirect Evidence** | **Imprecision** | **Other Considerations** | **Plantar orthosis** | **In plantar ortesis** | **Relative(95% CI)** | **Absoluto(95% CI)** |  |  |
| **Promotion of functionality and reduction of disability associated with the foot through orthopedological plantar support.** | | | | | | | | | | | | |
| 3 | Randomised trials | It's not serious | Serious | It's not serious | It's not serious | None | 84/170 (49.4%) | 86/170 (50.6%) | Not Estimable |  | ⨁⨁⨁◯Moderate |  |

**Biography**

1) BH, Mengshoel AM. Effect of a thin customized insole on pain and walking ability in rheumatoid arthritis: A randomized study. Musculoskeletal Care. 2018.

2) Gaino JZ, Bértolo MB, Nunes CS, Barbosa CM, Landim SF, Sachetto Z, Magalhães EP. The effect of foot orthoses on balance, foot function, and mobility in rheumatoid arthritis: A randomized controlled clinical trial. Clin Rehabil. 2021.

3) Reina-Bueno M, Vázquez-Bautista MDC, Pérez-García S, Rosende-Bautista C, Sáez-Díaz A, Munuera-Martínez PV. Effectiveness of custom-made foot orthoses in patients with rheumatoid arthritis: a randomized controlled trial. Clin Rehabil. 2019 Apr; 33(4):661-669. 2018.
