# Supplementary material for: A clinical practice guideline for the management of the foot and ankle in rheumatoid arthritis
Source: Rheumatol Int. 2024 Jun 8;44(8):1381–93. doi: 10.1007/s00296-024-05633-1 (PMC11222212; doi:10.1007/s00296-024-05633-1)
Supplement: Supplementary file 4 — Supplementary Material 14 [file 296_2024_5633_MOESM14_ESM.docx]

## Annex 4. GRADE Surgery Assessment

**Question:** Foot and ankle surgery in patients with rheumatoid arthritis.

| **Certainty assessment** | | | | | | | **No. of patients** | | **Effect** | | **Certainty** | **Importance** |
| --- | --- | --- | --- | --- | --- | --- | --- | --- | --- | --- | --- | --- |
| **No. of studies** | **Study Design** | **Risk of bias** | **Inconsistency** | **Indirect Evidence** | **Imprecision** | **Other Considerations** | **Surgery** | **No Surgery** | **Relative(95% CI)** | **Absoluto(95% CI)** |  |  |
| **Correction of osteoarticular deformation by means of invasive techniques.** | | | | | | | | | | | | |
| 13 | Observational studies | Serious | Serious | Serious | Serious | Strong association: all possible residual confounders could reduce the demonstrated effect | 817 Participants  - |  | Not Estimable |  | ⨁◯◯◯Very low |  |
|  | | | | | | | | | | | | |
| 1 | Randomised trials | It's not serious | Serious | Serious | Very serious | All possible residual confounders could reduce the demonstrated effect | 10/23 (43.5%) | 13/23 (56.5%) | Not Estimable |  | ⨁◯◯◯Very low |  |

**Bibliography:**

1.Donegan, R.J., Blume, P.A.. Functional Results and Patient Satisfaction of First Metatarsophalangeal Joint Arthrodesis Using Dual Crossed ScrewFixation. J. Foot Ankle Surg.; 2017 .

2.Fazal, M.A., Wong, J.H.M., Rahman, L. First metatarsophalangeal joint arthrodesis with two orthogonal two hole plates. FOOT & ANKLEINTERNATIONAL; 2018.

3.Pedersen, E., Pinsker, E., Younger, A.S., Penner, M.J., Wing, K.J., Dryden, P.J., Glazebrook, M., Daniels, T.R. Outcome of Total Ankle Arthroplasty inPatients with Rheumatoid Arthritis and Noninflammatory Arthritis. J. Bone Jt. Surg.; 2014.

4.Schrier JC, Keijsers NL,Matricali GA,Verheyen CCPM,Louwerens JWK. Resection or preservation of the metatarsal heads in rheumatoid forefootsurgery? A randomised clinical trial.. Foot Ankle Surg; 2019.

5.Kosuke Ebina, Makoto Hirao,Jun Hashimoto,Akihide Nampei,Kenrin Shi,Tetsuya Tomita,Kazuma Futai,Yasuo Kunugiza,Takaaki Noguchi & HidekiYoshikawa. Comparison of a self-administered foot evaluation questionnaire (SAFE-Q) between joint-preserving arthroplasty and resectionreplacementarthroplasty in forefoot surgery for patients with rheumatoid arthritis. Modern Rheumatology; 2016

6.Mainudden Bhavikatti∗, Mathew David Sewell,Nawfal Al-Hadithy,Sarfraz Awan,M.A. Bawarish. Joint preserving surgery for rheumatoid forefootdeformities improves pain and corrects deformity at midterm follow-up. The Foot; 2011.

7.Masahiro Horita, MD1,Keiichiro Nishida,MD,PhD1,Kenzo Hashizume,MD,PhD2,Yoshihisa Nasu,MD,PhD3,Kenta Saiga,MD,PhD1,RyuichiNakahara,MD,PhD4,Takahiro Machida,MD1,Hideki Ohashi,MD1,and Toshifumi Ozaki,MD,PhD1. Outcomes of Resection and Joint-Preserving Arthroplastyfor Forefoot Deformities for Rheumatoid Arthritis. Foot & Ankle International; 2018.

8.Anna Clara Benoni 1, Ann Bremander 2,3,and Anna Nilsdotter. Patient-reported outcome after rheumatoid arthritis-related surgery in the lowerextremities. Acta Orthopaedica ; 2012.

9.Laurence Dodd, MBBS,BSc,MRCS 1,Mordicai Atinga,MBBS,MRCS 1,Julian Foote,MBBS,MRCS 1,Simon Palmer,MBBS,FRCS-Tr Orth. Outcomes after theStainsby Procedure in the Lesser Toes: An Alternative Procedure for the Correction of Rigid Claw Toe Deformity. The Journal of Foot & Ankle Surgery;2011.

10.S. Thomas, Kinninmonth,MB,C. Senthil Kumar.. Lpng-term results of the modified hoffman procedure in the rheumatoid forefoots. THE JOURNAL OFBONE AND JOINT SURGERY; 2005.

11.Dieter Rosenbaum, PhD, Britta Timte, MD, Andreas Schmiegel, PhD, Rolf K. Miehlke, MD, Arvid Hilker, MD. First Ray Resection Arthroplasty VersusArthrodesis in the Treatment of the Rheumatoid Foot. Foot & Ankle International; 2011.

12.Jun-ichi Fukushi, Yasuharu Nakashima,Ken Okazaki,Hisakata Yamada,Taro Mawatari,Masanobu Ohishi,Akiko Oyamada,Yukio Akasaki,YukihideIwamoto,..abhiboneka bbazi Outcome of Joint-Preserving Arthroplasty for Rheumatoid Forefoot Deformities. Foot & Ankle International; 2015.

13.Edwin P. Su, , Barbara Kahn, , Mark P. Figgie, . Total Ankle Replacement in Patients with Rheumatoid Arthritis. CLINICAL ORTHOPAEDICS ANDRELATED RESEARCH; 2004.

14.Huub J L van der Heide, Bernard Schutte, Jan Willem K Louwerens, Frank H J van den Hoogen, and Maarten C de Waal Malefijt. Total ankle prosthesesin rheumatoid arthropathy. Acta Orthopaedica; 2009.
