# Supplementary material for: A clinical practice guideline for the management of the foot and ankle in rheumatoid arthritis
Source: Rheumatol Int. 2024 Jun 8;44(8):1381–93. doi: 10.1007/s00296-024-05633-1 (PMC11222212; doi:10.1007/s00296-024-05633-1)
Supplement: Supplementary file 5 — Supplementary Material 15 [file 296_2024_5633_MOESM15_ESM.docx]

## Annex 5. GRADE Self-Care Assessment

**Question: Foot** self-care for rheumatoid arthritis patients.

| **Certainty assessment** | | | | | | | **No. of patients** | | **Effect** | | **Certainty** | **Importance** |
| --- | --- | --- | --- | --- | --- | --- | --- | --- | --- | --- | --- | --- |
| **No. of studies** | **Study Design** | **Risk of bias** | **Inconsistency** | **Indirect Evidence** | **Imprecision** | **Other Considerations** | **Self-care DE** | **Control Not SE** | **Relative(95% CI)** | **Absoluto(95% CI)** |  |  |
| Normalization of foot self-care in RA. | | | | | | | | | | | | |
| 1 | Randomised trials | Serious | It's not serious | It's not serious | Serious | None | 56/202 (27.7%) | 148/202 (73.3%) | Not Estimable |  | ⨁⨁◯◯Low |  |
|  | | | | | | | | | | | | |
| 3 | Observational studies | Serious | Serious | Serious | Serious | All possible residual confounders could reduce the demonstrated effect | 293/293 (100.0%) |  | Not Estimable |  | ⨁◯◯◯Very low |  |

**Bibliography:**

1) Susann Arvidsson, Stefan Bergman,Barbro Arvidsson,Bengt Fridlund & Pia Tingström. Effects of a self-care promoting problem-based learning programme in people with rheumatic diseases: a randomized controlled study. JOURNAL OF ADVANCED NURSING; 2012.

2) R. Semple, L.W. Newcombe, G.L. Finlayson BSc Hons1, C.R. Hutchison BSc Hons1,J.H. Forlow BSc Hons1 and J. Woodburn PhD,MPhil,BSc Hons The FOOTSTEP self-management foot care programme: Are rheumatoid arthritis patients physically able to participate?. Musculoskelet. Care ; 2008.

3) Stolt, Minna, Kilkki, Mia, Suhonen., Riitta. Self-assessed foot health in older people with rheumatoid arthritis—A cross-sectional study. International Journal of Older People Nursing; 2020

4) Wang, Su-Yen, Chen, Hsiu-Hung. The Relationship Between Physical Function, Knowledge of Disease, Social Support and Self-Care Behavior in Patients With Rheumatoid Arthritis. Journal of Nursing Research; 2007.
