# Supplementary material for: A clinical practice guideline for the management of the foot and ankle in rheumatoid arthritis
Source: Rheumatol Int. 2024 Jun 8;44(8):1381–93. doi: 10.1007/s00296-024-05633-1 (PMC11222212; doi:10.1007/s00296-024-05633-1)
Supplement: Supplementary file 6 — Supplementary Material 16 [file 296_2024_5633_MOESM16_ESM.docx]

## Annex 6. GRADE Ulcer Management Assessment

**Question:** Management of RA ulcers.

| **Certainty assessment** | | | | | | | **No. of patients** | | **Effect** | | **Certainty** | **Importance** |
| --- | --- | --- | --- | --- | --- | --- | --- | --- | --- | --- | --- | --- |
| **No. of studies** | **Study Design** | **Risk of bias** | **Inconsistency** | **Indirect Evidence** | **Imprecision** | **Other Considerations** | **Management of RA ulcers** | **Comparison** | **Relative(95% CI)** | **Absoluto(95% CI)** |  |  |
| **Care of dermal lesions associated with osteoarticular deformation in the foot.** | | | | | | | | | | | | |
| 2 | Observational studies | Very serious | Serious | Serious | Serious | None | 273/416 (65.6%) | 136/416 34,4% | Not Estimable |  | ⨁◯◯◯Very low |  |

**Bibliography:**

1) Wilson O, Hewlett S, Woodburn J, Pollock J, Kirwan J. Prevalence, impact and care of foot problems in people with rheumatoidarthritis: results from a United Kingdom based cross-sectional survey. J Foot Ankle Res.2017.

(2) Bastidas Soria, Jacqueline Beatriz, Suárez Toledo, María Fernanda, Verónica Maribel Hurtado Hurtado, Calderón Martínez, Nathali Viviana. Cuban Journal of Rheumatology: RCuR, Vol. 20, No.1, 2018.
