# Supplementary material for: A clinical practice guideline for the management of the foot and ankle in rheumatoid arthritis
Source: Rheumatol Int. 2024 Jun 8;44(8):1381–93. doi: 10.1007/s00296-024-05633-1 (PMC11222212; doi:10.1007/s00296-024-05633-1)
Supplement: Supplementary file 7 — Supplementary Material 17 [file 296_2024_5633_MOESM17_ESM.docx]

## Annex 7. GRADE Evaluation Assessment

**Question:** Infiltrations in joints and tendons in the foot and ankle

| **Certainty assessment** | | | | | | | **No. of patients** | | **Effect** | | **Certainty** | **Importance** |
| --- | --- | --- | --- | --- | --- | --- | --- | --- | --- | --- | --- | --- |
| **No. of studies** | **Study Design** | **Risk of bias** | **Inconsistency** | **Indirect Evidence** | **Imprecision** | **Other Considerations** | **Infiltration of foot and ankle joints** | **Other disease, joint, and/or treatment.** | **Relative(95% CI)** | **Absoluto(95% CI)** |  |  |
| **Infiltration of corticosteroids into foot joints** | | | | | | | | | | | | |
| 2 | Observational studies | Serious | Serious | Serious | Serious | Publication bias is strongly suspected. | 96/108 (88.9%) | 12/108 (11.1%) | Not Estimable |  | ⨁◯◯◯Very low |  |
| **Infiltration in tendon in the foot** | | | | | | | | | | | | |
| 1 | Observational studies | Serious | Serious | Serious | Serious | None | 34/96 (35.4%) | 58/96 (60.4%) | Not Estimable |  | ⨁◯◯◯Very low |  |

**Bibliography:**

Maria-Antonietta d'Agostino; Xavier Ayral; Gabriel Baron; Philippe Ravaud; Maxime Breban; Maxime Dougados (2005). Impact of ultrasound imaging on local corticosteroid injections of symptomatic ankle, hind-, and mid-foot in chronic inflammatory diseases, 53, 284–292.

Furtado RNV, Machado FS, Luz KR da, Santos MF Dos, Konai MS, Lopes RV, et al. Intra-articular injection with triamcinolone hexacetonide in patients with rheumatoid arthritis: prospective assessment of goniometry and joint inflammation parameters. Rev Bras Reumatol. 2017; 57(2):115–21.

Macarrón Pérez, Pilar; Morales Lozano, María del Rosario; Vadillo Font, Cristina; Abásolo Alcázar, Lidia; Martínez Rincón, Carmen; Fernandez Gutierrez, Benjamin; Blanco Hontiyuelo, Margarita; González-Fernández, María Luz. Multidisciplinary approach in the treatment of tendinous foot involvement in rheumatoid arthritis. Clinical Rheumatology, 2021. 40,12:4889-4897.
