# Supplementary material for: A clinical practice guideline for the management of the foot and ankle in rheumatoid arthritis
Source: Rheumatol Int. 2024 Jun 8;44(8):1381–93. doi: 10.1007/s00296-024-05633-1 (PMC11222212; doi:10.1007/s00296-024-05633-1)
Supplement: Supplementary file 8 — Supplementary Material 18 [file 296_2024_5633_MOESM18_ESM.docx]

## Annex 8. Bibliographic searches carried out in the different databases

| Theme | Literature search |
| --- | --- |
| Chiropody | (rheumatoid arthritis) AND (callus OR corn OR hyperqueratosis) AND (nails OR foot OR forefoot OR feet) |
| Footwear | (rheumatoid arthritis) AND (footwear OR shoe* OR boot* OR deck OR trainer* OR sneacker*)  (rheumatoid arthritis[Title]) AND ((footwear OR shoe* OR boot* OR deck OR trainer* OR sneacker*)) |
| Plantar orthosis | (“foot”[MeSH Terms] OR “foot”[All Fields]) OR (“ankle”[MeSH Terms] OR “ankle”[All Fields] OR “ankle joint”[MeSH Terms] OR (“ankle”[All Fields] AND “joint”[All Fields]) OR “ankle joint”[All Fields])) AND rheumatoid arthritis[Title] AND ((“foot orthoses”[MeSH Terms] OR (“foot”[All Fields] AND “orthoses”[All Fields]) OR “foot orthoses”[All Fields] OR (“foot”[All Fields] AND “orthosis”[All Fields]) OR “foot orthosis”[All Fields]) OR insole[All Fields] OR (support[All Fields] AND plantar[All Fields])). |
| Surgery | (Rheumatoid Arthritis) AND (Foot OR Feet OR Ankle OR Bones of lower extremity OR Hallux OR First Metatarsophalang*) AND (Surgic* OR "non-conservative treatment") AND (Pain OR Disab* OR Funct*) |
| Self-care | ((Rheumatoid arthritis) AND (self care OR self-care) AND (feet OR foot)) not diabetic |
| Ulcers | “Reumatoid arthritis” AND “foot lesions”  “Ulcers” AND “ankle”  “Artritis reumatoide” AND “úlceras”  "Rheumatoid Arthritis" AND "Skin Lesions"  (Reumatoid arthritis) AND (“foot" [MeSH Terms] OR “foot” [All Fields] OR “anckle" [MeSH Terms] OR “anckle” [All Fields]) AND (“Skin lesions” [MeSH Terms] OR “skin lesions” [All Fields] OR "Ulcer*" [MeSH Terms] OR “Ulcer*” [All Fields]) |
| Physical therapy |  |
| Infiltrations | “Rheumatoid Arthritis” AND “glucocorticoids”[All Fields] OR “glucocorticoids”[MeSH Terms] OR Glucocorticoids[Text Word] OR “triamcinolone”[MeSH Terms] “hyaluronic acid”[MeSH Terms] OR Hyaluronic Acid[Text Word] “viscosupplements”All Fields] OR “viscosupplements”MeSH Terms] OR Viscosupplements[Text Word] - Platelet-Rich Plasma - Injections, Intra-Articular - “Intraarticular glucocorticoids” - Injections, Intralesional - Foot |
