# Supplementary material for: A clinical practice guideline for the management of the foot and ankle in rheumatoid arthritis
Source: Rheumatol Int. 2024 Jun 8;44(8):1381–93. doi: 10.1007/s00296-024-05633-1 (PMC11222212; doi:10.1007/s00296-024-05633-1)
Supplement: Supplementary file 9 — Supplementary Material 19 [file 296_2024_5633_MOESM19_ESM.docx]

Annex 9. Patient material and recommendations, degree of evidence and degree of agreement.

|  | Recommendations | GRADE Level of Evidence | Degree of agreement |
| --- | --- | --- | --- |
| **Chiropody** | Chiropodies are recommended for the removal of hyperkeratotic (helomas and thylomas) and nail lesions. | Very low | 9 [8-10] |
|  | Chiropodies should be completed with orthopaedic treatments or orthopaedic footwear. | Very low | 9 [9-10] |
| **Footwear** | Standardized therapeutic footwear benefits patients with RA by reducing pain and improving physical functionality, compared to store-bought footwear | High | 9 [7-10] |
|  | Standardized therapeutic footwear provides benefits in foot functionality (reducing plantar pressure), foot pain, physical functionality, and quality of life | High | 9 [9-10] |
| **Foot orthosis** | Foot orthoses optimize the biomechanics and function of the foot, providing cushioning and unloading the structures of the foot. | High | 10 |
|  | Foot orthoses reduce foot pain, improve physical function and quality of life | High | 10 [9-10] |
|  | Custom foot orthoses reduce foot pain and functionality, balance, and quality of life. | High | 10 [9-10] |
| **Surgery** | Total ankle arthroplasty is recommended for patients with RA | Very low | 7 [5-10] |
|  | Arthrodesis of the 1st metatarsophalangeal joint and arthroplasty of the 2nd to 5th metatarsal heads are recommended | Very low | 6 [5-10] |
|  | Scarf's technique is positive in short-term correction of hallux abductus valgus | Very low | 6 [5-9] |
| **Self-care** | It is recommended to work with patients on the limiting factors for self-care with all the factors involved in the care of patients with RA. | Very low | 9 [9-10] |
| **Ulcer Management** | Care of skin ulcers, such as vascular ulcers with a high risk of infection, is recommended | Very low | 10 [9-10] |
| **Physical therapy** | Moderate physical exercise with limited supervision is advisable as long as we respect the intensity, frequency and appropriate duration | Expert opinion | 8 [9-10] |
|  | Supervised in-office physical exercise has positive effects on quality of life, physical functioning, and pain |  | 8 [6-10] |
| **Injections** | Corticosteroid injection with previous ultrasound information improves stiffness and physical function results compared to infiltration with clinical and radiographic data alone | Moderate evidence | 8 [5-10] |
|  | Corticosteroid injection with clinical and radiographic data alone is capable of improving pain and, to a lesser extent, stiffness and physical function | Moderate evidence | 8 [5-9] |
|  | In patients with ankle arthritis, an injection of thiamcinolone hexatonic corticosteroid is effective in terms of pain and inflammation | Low evidence | 7 [5-9] |
|  | In patients with RA and tendinitis in the foot, an injection of corticosteroids together with a podiatric-orthotic program is effective in terms of pain, function and ultrasound (Doppler) | Moderate evidence | 9 [5-9] |
